# SARS-CoV-2 and Risk to Food Safety

**DOI:** 10.3389/fnut.2020.580551

**Published:** 2020-11-02

**Authors:** Lucia E. C. M. Anelich, Ryk Lues, Jeffrey M. Farber, Valeria R. Parreira

**Affiliations:** ^1^Centre for Applied Food Sustainability- and Biotechnology, Central University of Technology, Bloemfontein, South Africa; ^2^Department of Food Science, University of Guelph, Guelph, ON, Canada

**Keywords:** SARS-CoV-2, COVID-19, food safety, food packaging, surfaces, survival, inactivation

## Abstract

The COVID-19 pandemic has brought about a consideration of our understanding of transmission of the causal agent, SARS-CoV-2 to humans and its potential effect on food safety and food security. The main routes of transmission are reported to be person-to-person, by respiratory droplets and to a lesser degree, by fomites. Concerns have been raised on the possibility of transmission via food and food packaging and whether the virus poses a risk to food safety. The current contribution provides an exposé of updated literature and reports applicable to various components of food safety and its linkage to SARS-CoV-2. The article focuses on SARS-CoV-2 survival in food, on food contact materials and food packaging, and its categorization as a foodborne vs. respiratory virus, the possibility of fecal-oral transmission and the likelihood of infection via the gastro-intestinal system. The survival and inactivation of SARS-CoV-2 in food through thermal and non-thermal inactivation methods as well as the survival and inactivation on inanimate surfaces and effective disinfection of food contact surfaces, are discussed. Ultimately, the article endeavors to add to the body of knowledge pertaining to the role of SARS-CoV-2 in food safety and thereby contribute to an agile and robust fraternity that is equipped to absorb and weather the ongoing effects of the pandemic on the food sector.

## Introduction

COVID-19 is caused by the SARS-CoV-2 virus (Severe Acute Respiratory Syndrome Coronavirus 2)—a member of the *Coronaviridae* Family generally comprising an enveloped, single-stranded RNA structure. A distinguishing characteristic of the *Coronaviridae* is the club- or petal-shaped surface projections or “spikes” which is reminiscent of the solar corona, hence the name coronaviruses (1). The virus particle of SARS-CoV-2 ranges from 50 to 200 nm and comprises a Spike (S), Envelope (E), Membrane (M), and Nucleocapsid (N) structural proteins, the latter containing the genome (RNA). The S, E, and M proteins constitute the viral envelope (2, 3).

Coronaviruses mostly infect amphibians, birds, and mammals, with a number shown to be zoonotic (4). Four coronavirus genera (α, β, γ, and δ) have been identified so far, however, in the last 2 decades, three novel β coronaviruses (MERS-CoV, SARS-CoV-1, and SARS-CoV-2), now known to cause respiratory-related disease outbreaks in humans have been described, although each has displayed unique features (5). Although SARS (Severe Acute Respiratory Syndrome) and MERS (Middle-East Respiratory Syndrome) have significantly higher case-fatality rates than SARS-CoV-2, the latter is notably more infectious and spreads readily among humans. Table 1 provides a comparison of the fundamental traits of the post-2000 coronavirus outbreaks (8–10).

**Table 1:** A comparison of key indicators amongst post-2000 coronavirus outbreaks.

**Disease**	**SARS**	**MERS**	**COVID-19**
Virus species	SARS-CoV-1	MERS-CoV	SARS-CoV-2
Origin	2002, China's Guangdong province	2012, Saudi Arabia	2019, Wuhan, China
Natural reservoir	Horseshoe bat (*Rhinolophus affinis*)	Horseshoe bat (*Rhinolophus affinis*)	Horseshoe bat? (*Rhinolophus affinis*)
Intermediate host	Civet cats	Dromedary camels	Pangolins?
Transmission	Droplets produced by coughing, sneezing, talking, or breathing	Relatively low, person-to-person droplets in close family or healthcare circles	Highly infectious, person -to-person via droplets produced by coughing, talking, breathing, and sneezing; degree of transmission via surfaces
Mean incubation period	ca. 5 days	5.2 days	5–6 days
Main symptoms	Cough (dry at first), fever, and diarrhea	Fever, cough, shortness of breath	Fever, dry cough, tiredness, sore throat, loss of smell, and taste
Case fatality rate	9.6–15%	34.3%	1.7–12.9% country distribution with a world average of 3.3%^b^
R${}_{0}^{\text{a}}$	Initial above 2; settled at <1 as pandemic developed	Initially above 2; settled at 0.45 as pandemic developed	1.5–3.5 or even higher (current global mean estimates between 2 and 3)

a *Basic Reproductive number (R0): The number of cases that are expected to occur on average in a homogeneous population as a result of infection by a single individual, when the population is susceptible at the start of an epidemic, before widespread immunity starts to develop and before any attempt has been made at immunization (6)*.

b *As per the European CDC figures at the time of publication (7). This figure is likely to change as the pandemic progresses*.

Data from various epidemiological studies report that COVID-19 is transmitted amongst humans mainly through respiratory droplets (breathing, sneezing, coughing, talking) or via the contact-oral route with contaminated objects and surfaces, although the latter seems to be less-significant (11, 12). A characteristic of SARS-CoV-2 infections that has played a pivotal role in the epidemiological spread is its ability to shed infective particles in the absence of symptoms. This is categorized as (1) after infection, but prior to showing symptoms (pre-symptomatic); (2) during infection, but not showing any symptoms (asymptomatic); and (3) after having recovered, but still shedding virulent particles (post-symptomatic). Research has indicated that the pre-symptomatic shedding (infective) window of COVID-19 typically ranges from 2 to 3 days after initial infection, followed by the asymptomatic phase over ca. 4–10/11 days and the post-symptomatic phase thereafter (from 11 up to 14 days or until the individual test is negative) (13).

In addition to the symptoms mentioned in Table 1, secondary complications of advanced COVID-19 disease can include breathing difficulty, chest pain or pressure, confusion, inability to stay awake, nausea, vomiting, diarrhea, and oxygen deprivation indicated by bluish lips or face. Ultimately, mortality manifests in an elevated immune response causing a cytokine over-production and severe pneumonia (14, 15). A number of co-morbidities and underlying health problems have been reported to impact the severity of the disease, amongst which are age, individuals suffering from chronic lung disease and asthma, heart conditions, hypertension, diabetes, obesity, and immunocompromised health (16). In children, the disease may manifest as a multisystem inflammatory syndrome, which can lead to serious and life-threatening consequences in previously healthy children and adolescents (17).

The mentioned underlying conditions are notably augmented in poor and under-developed communities, mostly due to compromising health practices, limited awareness and knowledge, lacking resources, and healthcare. The urban poor and rural residents, migrants, informal workers, those living in conflict-prone areas and individuals with co-morbidities have been identified as particularly at-risk (18). According to Guan and Liang (19), patients with comorbidities of over and under nutrition, hypertension and diabetes and those having malignancy yielded poorer clinical diagnoses than without. The observation that COVID-19 is especially fatal to individuals experiencing chronic or acute hunger or malnourishment constitute a particular risk in Africa with undernourishment affecting ca. 20% of the population—the highest incidence globally (20–22).

Amongst vulnerable groups, deficient water, sanitation, and housing infrastructure have presented a significant concern in light of the COVID-19 pandemic, as the provision of clean running water is a pivotal element of basic hygiene, especially considering proper WASH (water, sanitation, and hygiene) as the first line of defense against COVID-19 transmission. Currently, about 40% of people globally do not have basic handwashing facilities at home (23). Similarly, the densely populated domestic environment and ailing housing infrastructure eminent in squatter camps and informal settlements where one- or two-bedroom shacks are often occupied by 6 or more individuals, obviously challenges the principle of maintaining social distancing. The disruptive effect on health service delivery and the consequences for diseases such as malaria, tuberculosis (TB), and human immunodeficiency virus (HIV) in poor societies has also been a growing concern. By posing additional demands to already stretched healthcare systems, unexpected epidemics such as those caused by MERS and SARS-CoV-1 have shown to significantly undermine routine control efforts of TB, Malaria and HIV-AIDS, and have led to a considerable increase in, for example, malaria-related mortalities in a number of developing countries. Apart from resource concerns, the unique interaction between TB and HIV in that ca. 60% of TB patients are also HIV-positive, renders COVID-19 likely to be of particular concern for communities with high rates of TB and HIV, particularly where these illnesses are poorly controlled.

## Transmission of SARS-CoV-2 by Food and Food Packaging

Several risk assessments have been conducted by food safety agencies on whether food, food contact materials and food packaging present a potential risk to food safety related to SARS-CoV-2. Considering that much was unknown about SARS-CoV-2 and its survival in food, on food contact materials or food packaging when the pandemic struck, scientists turned to published information on similar viruses. These included the first SARS virus (SARS-CoV-1), MERS and coronaviruses that cause the common cold (24–28). Establishing conservative risk estimates is possible, even when presented with significant data gaps. For a risk assessment of this nature, an intensive scrutiny of literature is required, followed by interrogation of that information to develop interim guidance for both policymakers and the public. As novel information emerges, such guidance is then updated accordingly.

According to authoritative literature, the consensus is that currently, there is no evidence that SARS-CoV-2 is a food safety risk. Therefore, from a hazard-risk perspective, the overall potential risk of acquiring COVID-19 from contaminated food or food packaging appears to be very low (27–33). SARS-CoV-2 is therefore, not considered a foodborne virus. It remains primarily a respiratory virus, which may also enter the bloodstream via mucous membranes in the eyes (34).

The French Agency for Food, Environmental and Occupational Health and Safety conducted one of the first risk assessments related to food safety and SARS-CoV-2 (27). Several questions were posed to the expert panel, one of which was whether there was a likelihood of contracting COVID-19, from ingesting the virus. Zhang et al. (35) found viral genetic material in anal swabs and blood taken from 178 patients. Considering that one of the symptoms of COVID-19 is diarrhea, these matters raised concerns on the likelihood of the virus being transmitted via the fecal-oral route. However, there are no reports to date showing fecal-oral transmission of the virus (36). A key question to address is how well SARS-CoV-2 survives during passage through the human stomach. It is known that SARS-CoV-2 uses angiotensin- converting enzyme 2 (ACE) as receptors, which are expressed on bronchial epithelial cells, mucosal, and epithelial cells in the intestine (37); however, we do not know the concentration of SARS-CoV-2 necessary to infect humans via the gastrointestinal route and whether SARS-CoV-2 can actually invade these cells and eventually enter into the bloodstream and/or if diarrhea is a main symptom. Lamers et al. (38) reported infection of enterocytes in human small intestine organoids by both SARS-CoV-1 and SARS-CoV-2 during *in vitro* studies and stated that SARS-CoV-2 replication was supported by intestinal epithelium. However, several studies have concluded that diarrhea is likely caused when the virus infiltrates the body through other mechanisms, during the severe stage of the illness and not to the virus being contracted from ingestion of contaminated food, via the gastro-intestinal tract (39–42). A further consideration affecting the likelihood of infection via the gastro-intestinal system, is that virus in food is likely to be at low concentration and may also be less available to host cells when contained within a food matrix in comparison to the virus being carried in respiratory droplets (43).

Food manufacturing businesses implement Food Safety Management Systems (FSMS), albeit at different levels of sophistication, depending on requirements in different countries. In all cases, however, development and implementation of a successful FSMS requires a solid foundation of Good Hygiene Practices (GHP). Such practices include washing hands with soap and water for a minimum of 20 s at specific times. In several cases, sanitizing hands is required after hand washing. There are additional hygienic practices listed in all such standards to ensure that food handlers practice proper hygiene to prevent transmission of any potential microbiological contamination to food (44).

## Survival and Inactivation of SARS-CoV-2 in Food

Thermal (e.g., heat) and non-thermal (e.g., radiation and ultrasound) inactivation methods can be used to inactivate or reduce pathogens, including viruses, in the environment and on food (45, 46). Different thermal treatments have been used for the inactivation of foodborne viruses (e.g., human norovirus, hepatitis A and E viruses) on food matrices or liquids (47). Dry (hot air oven or incineration) and humid heat (steam, autoclave) are very effective methods for inactivating viruses and bacteria (45, 47, 48). After conducting an analysis of 10 different studies, Kampf et al. (48), reported that five different types of coronavirus suspended in liquid media, including SARS-CoV-1 and MERS-CoV, could be reduced by at least 4 logs using thermal treatments such as 60°C for 30 min, 65°C for 15 min or 80°C for 1 min. Chin et al. (49) found that SARS-CoV-2 was reduced by about 7 logs after a heat treatment of 70°C for 5 min. Furthermore, ANSES (27) considered the matter of sufficient heat treatment of food (27) and concluded that exposure of food to 63°C for 4 min would be adequate to kill the virus. In a qualitative risk assessment conducted by the Food Standards Agency of the United Kingdom, the organization acknowledged that despite several uncertainties, temperatures used for cooking should be sufficient to inactivate any virus present in food (28).

At refrigeration temperatures (4°C), Rabenau et al. (50) found no loss of infectious titer for SARS-CoV-1. A similar resistance to refrigeration was found by Chin et al. (49) when hardly any reduction in infectious SARS-CoV-2 was noted in transport medium held at 4°C for 14 days, which was the length of the experiment. It is therefore likely that the virus would survive for longer periods at refrigeration temperatures in specific media. Considering that freezing is generally not regarded as a destruction method for viruses in food, but rather as a method of preservation, it is likely that SARS-CoV-2 would survive freezing. In this regard, Fisher et al. (51) found that infectious SARS-CoV-2 did not decline in titer and was able to survive for 3 weeks in inoculated pieces of chicken, pork and salmon stored at 4, −20, and −80°C. Furthermore, Mullis et al. (52) demonstrated that a bovine coronavirus present on lettuce stored at 4°C retained its infectivity for at least 14 days. In addition, coronavirus 229E survived well for 2 days on lettuce stored at 4°C, before quickly decreasing in numbers, but did not survive on the surface of strawberries, potentially because of the acidity (53).

SARS-CoV-2 appears to be stable at different pH values (3–10) at room temperature, however, alkaline pH (>12) or acidic pH (<3) as well as heat, sunlight and UV light appear to be capable of inactivating the virus (49, 54, 55). Alternative methods of non-thermal physical disinfection include (i) ultraviolet (UV) light, (ii) pulsed light (iii) ionizing radiation, (iv) high pressure, (v) cold plasma, and (vi) high-intensity ultrasound (56), with some of these treatments limited to disinfecting surfaces.

Cold plasma treatment is a relatively new disinfection treatment that has attracted attention due to it being environmentally friendly, i.e., chemical free (57). This technology uses different inert gases that when submitted to high electricity, generate a large amount of a mixture of electrons, charged atoms and neutral atoms, which have the potential to inactivate microorganisms (57). The cold plasma technique uses cold gases to disinfect food contact surfaces as well as liquid and solid food products (56). Although it has been shown that cold plasma can effectively inactivate pathogenic viruses (e.g., human norovirus, adenovirus, and hepatitis A virus) on or in various matrices (57), further research is required to evaluate its effectiveness against SARS-CoV-2.

## Survival and Inactivation of SARS-CoV-2 on Inanimate Surfaces

It is well-established that viruses causing respiratory infections, particularly SARS-CoV-2, can be transmitted by indirect contact (fomites) through the environment (58, 59), particularly when one touches contaminated surfaces and subsequently touches one's mouth, nose or eyes, without first washing hands. Warnes et al. (60), showed that human coronavirus 229E (HuCoV-229E), which is closely related to SARS-CoV-2, was able to persist on the surface of 5 different materials [e.g., polytetrafluoroethylene (Teflon), polyvinyl chloride (PVC), ceramic tiles, glass, and silicone rubber] for at least 5 days. In a review conducted by Kampf et al. (61) on the persistence of coronaviruses on inanimate surfaces, SARS-CoV-1, MERS coronavirus or the endemic human coronavirus (HuCoV-229E), persisted on inanimate surfaces like metal, glass or plastic for up to 9 days. At 30°C or greater, the duration of persistence of MERS was shorter, whereas persistence of the transmissible gastroenteritis virus (TGEV) on surfaces increased to over 28 days at 4°C. More recent research has shown that in an experimental setting at between 21 and 23°C at 40% humidity, SARS-CoV-2 could remain viable for up to 72 h on plastic and stainless steel, up to 4 h on copper, and up to 24 h on cardboard (12). Pezzotti et al. (62) demonstrated that silicon nitride can inactivate 99% of the SARS-CoV-2 virus after exposure for 1 min, which showed it to be as effective as copper. Therefore, the use of Si_3_N_4_ particles placed into the fabric of personal protective equipment, as well as other material, could be an effective way to decrease viral spread. In addition, Ratnesar-Shumate et al. (63) showed that SARS-CoV-2 is sensitive to artificial sunlight when suspended in simulated saliva on stainless steel coupons, i.e., 90% of the virus was inactivated in ~7 min.

Chin et al. (49) found in an experimental setting, that no infectious virus was recovered from a number of surfaces after different times at 22°C. Notably, the virus was more stable on smooth surfaces with no infectious virus detected after 4 days on glass and banknotes and none detected after 7 days on plastic and stainless steel. However, it is important to note that such results were generated under experimental conditions and may not reflect the potential of picking up the virus from contact in a more realistic environment. The CDC (64) and other similar agencies and organizations (65), do not consider contracting SARS-CoV-2 via contaminated surfaces a main route of transmission. A report by the McKinsey Company seems to support this belief (66). This report states that approximately 90% of SARS-CoV-2 transmissions occur from symptomatic, pre-symptomatic and asymptomatic people, leaving 10% transmissions from the environment, which includes surfaces. Therefore, the greatest risk remains person-to-person transfer in a food environment, including manufacturing, retail and food service. This emphasizes the importance of wearing appropriate Personal Protective Equipment (PPE) and practicing proper hand hygiene and social distancing.

## Disinfecting Food Contact Surfaces

Food contact surfaces include all areas that come into contact with food products during preparation (e.g., cutting boards, tables, utensils), production, processing and packaging and typically include stainless steel, plastic material, wood, rubber, ceramics, or glass (54, 60, 67). These surfaces could be contaminated with pathogenic bacteria and viruses, which can infect the food and/or people handling the food (68). Even though current consensus is that SARS-CoV-2 is not transmitted by food of food packaging material, it is very important to properly clean and sanitize food contact surfaces, since one of the modes of transmission of SARS-CoV-2 appears to be touching contaminated surfaces and then touching your mouth, nose or eyes (28, 69, 70).

The current WHO guidance states that “thoroughly cleaning environmental surfaces with water and detergent and applying commonly used hospital level disinfectants (such as sodium hypochlorite) are effective and sufficient procedures” (58). Disinfectants are very important in the control and inactivation of microorganisms on various inanimate surfaces (61, 71, 72). However, if not careful, they can leave harmful residues behind on food contact surfaces.

The use of UV light is a well-known method for inactivating viruses, mycoplasma, bacteria and fungi, especially on surfaces (45, 46). In particular, UV-C light, the shortest wavelength (100–280 nm), has been largely used in the food industry (46). Several studies have demonstrated that UV-C light may be an effective tool for inactivating SARS-CoV-1 after a treatment of 60 min (73–76).

If surfaces are dirty, they should be cleaned using a detergent or soap and water, prior to disinfection. The food industry generally uses sanitizers that are considered safe due to low toxicity and non-corrosiveness (45). Sanitizers used on food contact surfaces should be different from those used on non-food contact surfaces (77, 78). Food grade sanitizers are able to reduce or control specific bacteria, while food grade disinfectants are able to kill bacteria, viruses, and molds, however, it is necessary to rinse with water to eliminate residues (77, 79).

Although the biology of SARS-CoV-2 is not fully understood, it belongs to the coronavirus family of enveloped viruses, which makes them susceptible to detergents and a variety of other microbicides (24), even more so than fungi, vegetative bacteria and yeasts (80). On environmental surfaces, studies have shown that 0.1% sodium hypochlorite, 0.5% hydrogen peroxide, and 62–71% ethanol can significantly reduce coronavirus presence on surfaces after 1 min of exposure at room temperature [(61); see Table 2]; similar effects have been seen with SARS-CoV-2 (49, 58). Clean water, at a temperature of at least 77°C, for at least 45 s can also be used. The Environmental Protection Agency (82), Health Canada (83), as well as the European Union Open Data Portal (84) have a list of approved disinfectants for use against COVID-19. SARS-CoV-2, like other viruses, cannot multiply in food, therefore, over time, the number of infectious virions is expected to decrease if the virus happens to be present on the surface of a food product (54). Air disinfection could be considered in food-related environments and two practical methods include room air cleaners such as filters or UV light, as well as upper-room germicidal UV fixtures (85).

**Table 2:** Inactivation of coronaviruses by different types of disinfecting agents.

**Disinfectants**	**Temperature/working concentration**	**Treatment time**	**Reduction of the virus titer**	**References**
Hot water	>75°C	>45 s-5 min	N/A**	(78)
Ethanol	60–71%	1 min	N/A*	(81)
Sodium hypochlorite	0.05% (500 ppm)	5 min	>3 log${}_{10}^{**}$	(78)
	0.1% (1,000 ppm)	5 min	>3 log${}_{10}^{**}$	(78)
Hydrogen peroxide	0.5%	1 min	>4 log${}_{10}^{***}$	(61)
Benzalkonium chloride	0.04%	1 min	<3 log${}_{10}^{***}$	(61)
Iodophor detergent	0.5–10%	~1 min	~3 log${}_{10}^{***}$	(77)

*SARS-CoV-1; **SARS-CoV-2; ***coronaviruses in general.

*N/A, not applicable*.

In summary, although the current evidence suggests that SARS-CoV-2 does not cause foodborne illness, the virus has caused major disruptions to the global food supply chain. Thus, issues such as food security and food sustainability have been brought to the forefront in this pandemic, as well as the health of food workers from farm through to retail and foodservice. Newer approaches/paradigms, such as the integration of One Health (e.g., creating a large team of specialists from many disciplines) along with systems thinking (i.e., a health crisis can rapidly spread to other systems that would normally appear unconnected), will be paramount to controlling pandemics in the future.

## Author Contributions

LA initiated and coordinated the article and wrote the sections on transmission of SARS-CoV-2 through food and food packaging as well as survival of the virus on surfaces. RL wrote the introduction, whilst VP authored the sections on disinfection of surfaces and inactivation of the virus in foods. JF contributed to all sections. All authors contributed to editing of the final article.

## Conflict of Interest

The authors declare that the research was conducted in the absence of any commercial or financial relationships that could be construed as a potential conflict of interest.
